# Extent of third-order linkage disequilibrium in a composite line of Iberian pigs

**DOI:** 10.1186/s12863-018-0661-4

**Published:** 2018-08-17

**Authors:** Luis Gomez-Raya, Luis Silio, Wendy M. Rauw, Luis Alberto Gracia-Cortés, Carmen Rodríguez

**Affiliations:** Instituto Nacional de Investigación y Tecnología Agraria y Alimentaria (INIA), Ctra. de La Coruña km 7, Madrid, 28040 Spain

**Keywords:** Third order linkage disequilibrium, Iberian pigs, High order linkage disequilibrium, Linkage disequilibrium

## Abstract

**Background:**

Previous studies on linkage disequilibrium have investigated second order linkage disequilibrium in animal and plant populations. The objective of this paper was to investigate the genome-wide levels of third order linkage disequilibrium in a composite line founded by admixture of four Iberian pig strains. A model for the generation of third order linkage disequilibrium by population admixture is proposed. A computer Expectation-Maximization algorithm is developed and applied to the estimation of third order linkage disequilibrium at inter- and intra-chromosomal level using 26,347 SNPs typed in 306 sows. The relationship of third order linkage disequilibrium with physical distance was investigated over 35 million triplets in SSC12. Basic and normalized estimates of inter and intra-chromosomal third order linkage disequilibrium are reported.

**Results:**

Genome-wide analyses revealed that third order linkage disequilibrium is rather common among linked loci in this Iberian pig line. It is shown that population admixture of multiple populations may explain the observed levels of third order linkage disequilibrium although it could be generated by genetic drift. Third order linkage disequilibrium decreases rapidly up to 4 Mb and then declines slowly. The short distances between consecutive markers explain the maintenance of the observed third order linkage disequilibria levels when using a model incorporating the break-up of disequilibrium by recombination. Genome-wide testing also revealed that only 3.6% of the normalized estimates were different from 1, − 1, 0, or from a not well-defined situation in which there is only one possible value for the third order linkage disequilibrium parameter, given allele frequencies and pairwise linkage disequilibria parameters.

**Conclusions:**

Third order linkage disequilibrium is common among linked markers in the analyzed pig line and may have been generated by population admixture of multiple populations or by genetic drift. As with second order linkage disequilibrium, the absolute value of the third order linkage disequilibrium decreases with physical distance. Normalization of third order linkage disequilibrium should be avoided for closely linked bi-allelic loci.

**Electronic supplementary material:**

The online version of this article (10.1186/s12863-018-0661-4) contains supplementary material, which is available to authorized users.

## Background

Linkage disequilibrium is defined as the non-random association of alleles at two or more loci. In many instances it is due to the physical organization of DNA sequences in which each nucleotide follows another in one single chain. It can also be due to genetic drift or selection. Linkage disequilibrium is a key parameter to understand evolution and as stated by Slatkin, it is an indicator of the population genetic forces that structure a genome [1, 2]. In the last decades, research on linkage disequilibrium has enhanced as an aid to map genes affecting diseases or quantitative traits in the so called Genome-Wide Associations Studies (GWAS; [3]), and in genomic selection aimed at exploiting associations of alleles at production traits with single nucleotide polymorphisms scattered over the entire genome [4]. There is a bulk of literature on these topics but the great majority of them consider just two loci at a time, however, the associations between alleles can occur for any number of loci. Third and higher order linkage disequilibrium is related to the association of alleles at several loci. Because of its complexity, high order linkage disequilibrium has not been much investigated in both animal or plant populations or in applications to help to understand their role in the expression of phenotypic traits. High order linkage disequilibrium might be related to epistasis because of its nature involving multiple loci. However, it is not a simple association of one allele to several alleles belonging to other loci but the association of one of the alleles to several haplotypes at the other loci. That is, third order linkage disequilibrium arises from the association between alleles not explained by second order linkage disequilibrium.

The classification and number of parameters characterizing linkage disequilibrium for any number of loci is given in Table 1. The number of haplotypes increases exponentially with the number of loci but the actual value of the high order parameter reduces also exponentially. The number of parameters to estimate (allele frequencies, second and higher order parameters) increases rapidly as the number of loci increase. Geiringer [5] and Bennet [6] described, first, higher order linkage disequilibria but their papers did not explain equations with enough detail and, consequently, applications were halted for a number of years. Thereafter, Thomson and Baur [7] described the relationship of haplotype frequencies with allele frequencies, second, and third order disequilibria parameters. They applied their method to an example using the HLA complex. Later on, a maximum likelihood framework with an Expectation-Maximization (EM) algorithm for estimation (similar to the two locus system algorithm of Excoffier and Slatkin, [8]) was developed for three and four multi-allelic loci by Long et al. [9]. However, Thomson and Baur [7] had realized that the second order linkage disequilibria set constraints on the third order parameter which complicated understanding the third order linkage disequilibrium parameter. Robinson et al. [10] proposed to normalize third order linkage disequilibrium after accounting for mutual constraints between second and third order parameters.

**Table 1:** Components of high order linkage disequilibrium

Number of loci	Maximum Order LD	Number of haplotypes	Max value of highest order disequilibrium	Number allele freq. to estimate	LD parameters to estimate
2	2	4	0.25	2	1
3	3	8	0.125	3	4
4	4	16	0.0625	4	11
n	n	2^*n*^	$\frac{1}{2^n}$	n	2^*n*^-*n-*1

All this research was carried out in the eighties and nineties when the development of genetic markers was still in its infancy (with microsatellites at their peak use) but with little coverage of animal genomes for today’s standards. The development of array technologies incorporating from thousands to hundreds of thousands of Single Nucleotide Polymorphisms (SNPs) has provided new tools to uncover the associations between alleles at different loci located elsewhere in the genome. Kim et al. [11] proposed a multi-locus high order linkage disequilibrium with a multiple order Markov chain model. Berg et al. [12] have developed a method for estimation of multi-allelic third order linkage disequilibrium. Nevertheless, no publication exists on the genome-wide levels of third order linkage disequilibrium present in animal populations.

The objective of this paper is to investigate third order linkage disequilibria using a 60 K SNP array of Illumina in a closed population of Iberian pigs. This is the first report on the extent of third order linkage disequilibria in animal genomes. In order to carry out extensive third order linkage disequilibrium estimation, a simple and efficient EM algorithm for the estimation of third order linkage disequilibrium of biallecic markers was also developed. In addition, the way that third order linkage disequilibrium is generated by population admixture was also investigated.

### Third order linkage disequilibrium theory

**Fig. 1:**
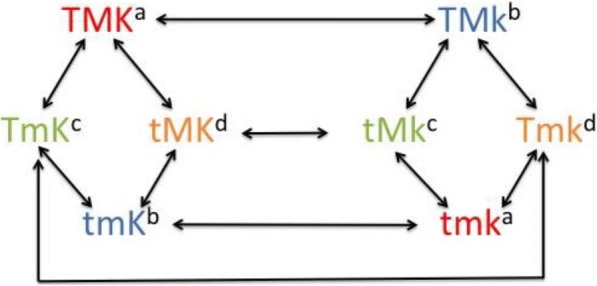
Configurations of all possible haplotypes for a three-locus system. Each arrow represents a one-time mutation in one of the SNPs (T/t, M/m, and K/k). Complementary haplotypes share the same color and superscript

Second order linkage disequilibrium in closely linked loci exists because one mutated allele is associated to a short stretch of an ancestral haplotype. Breaking down of associations by recombination erodes second order linkage disequilibrium but genetic drift can generate linkage disequilibrium again if populations are small. In addition, second order linkage disequilibrium exists because of co-selection of close or distant loci affecting quantitative traits. On the contrary, third order linkage disequilibrium cannot be generated itself by mutation between closely linked loci unless several haplotypes are lost by genetic drift. Figure 1 shows the eight possible haplotypes in a three locus systems. For third order linkage disequilibrium to exist, an association must occur such as allele *K* with haplotypes *TM* and *tm*, and allele *k* with haplotypes *Tm* and *tM.* For example, if the three loci are very closely linked (assuming no recombination) and each arrow between haplotypes represents a mutation then TMK, tmK, tMk, and Tmk must exist for full third order linkage disequilibrium. This requires two mutations (arrows) for creation of each haplotype and also the loss of intermediate haplotypes; for unlinked loci, a combination of haplotypes could also exist by the loss of the other haplotypes by either genetic drift or selection, which is accelerated by free recombination. Population admixture could facilitate the creation of third order linkage disequilibrium by disconnecting the processes of genetic drift and selection in the two populations. A population admixture model to generate third order linkage disequilibrium is introduced in the next section.

### Generation of third order linkage disequilibrium by population admixture

It is well established that the crossing between two populations differing in allele frequencies at two loci may generate second order linkage disequilibrium [13]. In this section, the generation of third order disequilibrium by admixture of two populations is shown. Let Z be the resulting cross from two populations, X and Y. Let three loci *T/t*, *M/m*, and *K/k* be located in that order on a chromosome. We will assume that these loci are not affected by selection.

The frequency of haplotype TMK at the cross Z is

1 $$ {f}_{TMK}^Z=\tau {f}_{TMK}^X+\left(1-\tau \right)\ {f}_{TMK}^Y $$

where *τ* is the mixing proportion of the two populations at crossing. The haplotype frequencies at the two populations at crossing can be put in terms of allele frequencies and second and third order linkage disequilibrium parameters:

2 $$ {f}_{TM K}^X={f}_T^X{f}_M^X{f}_K^{\mathrm{X}}+{f}_T^X{\delta}_{MK}^X+{f}_M^X{\delta}_{TK}^X+{f}_K^X{\delta}_{TM}^X+{\delta}_{TM K}^X $$

$$ {f}_{TM K}^Y={f}_T^Y{f}_M^Y{f}_K^Y+{f}_T^Y{\delta}_{MK}^Y+{f}_M^Y{\delta}_{TK}^Y+{f}_K^Y{\delta}_{TM}^Y+{\delta}_{TM K}^Y $$

where ${\delta}_{MK}^X$, ${\delta}_{TK}^X$, ${\delta}_{TM}^X$, are second order linkage disequilibrium parameters for loci MK, TK, and TM, respectively in population *X*; ${\delta}_{TMK}^X$is the third order linkage disequilibrium coefficient in population X*.* The same coefficients but with superscript Y are for population Y.

The allele frequencies at *T/t*, *M/m* and *K/k* in the crossed populations are given by:

3 $$ {\displaystyle \begin{array}{l}{f}_T^Z=\tau {f}_T^X+\left(1-\tau \right)\ {f}_T^Y\\ {}{f}_M^Z=\tau {f}_M^X+\left(1-\tau \right)\ {f}_M^Y\\ {}{f}_K^Z=\tau {f}_K^X+\left(1-\tau \right)\ {f}_K^Y\end{array}} $$

Then, the third order linkage disequilibrium in the crossed population, Z, is:

4 $$ {\delta}_{TM K}^Z={f}_{TM K}^Z-{f}_T^Z{f}_M^Z{f}_K^Z-{f}_T^Z{\delta}_{MK}^Z-{f}_M^Z{\delta}_{TK}^Z-{f}_K^Z{\delta}_{TM}^Z $$

After substituting eqs. (1), (2), and (3) into (4), the third order linkage disequilibrium in the cross  becomes:

5 $$ {\displaystyle \begin{array}{l}{\delta}_{TM K}^Z=\tau {f}_T^X{f}_M^X{f}_K^X+\tau {f}_T^X{\delta}_{MK}^X+\tau {f}_M^X{\delta}_{TK}^X+\tau {f}_K^X{\delta}_{TM}^X+{\tau \delta}_{TM K}^X\\ {}+\left(1-\tau \right){f}_T^Y{f}_M^Y{f}_K^Y+\left(1-\tau \right){f}_T^Y{\delta}_{MK}^Y+\left(1-\tau \right){f}_M^Y{\delta}_{TK}^Y+\left(1-\tau \right){f}_K^Y{\delta}_{TM}^Y+\left(1-\tau \right){\delta}_{TM K}^Y\ \\ {}-\left(\tau {f}_T^X+\left(1-\tau \right){f}_T^Y\right)\left(\tau {f}_M^X+\left(1-\tau \right){f}_M^Y\right)\left(\tau {f}_K^X+\left(1-\tau \right){f}_K^Y\right)\ \\ {}-\left(\tau {f}_T^X+\left(1-\tau \right){f}_T^Y\right)\ \left({\tau \delta}_{MK}^X+\left(1-\tau \right){\delta}_{MK}^Y+\tau \left(1-\tau \right)\left({\gamma}_M{\gamma}_K\right)\right)\\ {}-\left(\tau {f}_M^X+\left(1-\tau \right){f}_M^Y\right)\ \left({\tau \delta}_{TK}^X+\left(1-\tau \right){\delta}_{TK}^Y+\tau \left(1-\tau \right)\left({\gamma}_T{\gamma}_K\right)\right)\\ {}-\left(\tau {f}_K^X+\left(1-\tau \right){f}_K^Y\right)\ \left({\tau \delta}_{TM}^X+\left(1-\tau \right){\delta}_{TM}^Y+\tau \left(1-\tau \right)\left({\gamma}_T{\gamma}_M\right)\right)\end{array}} $$

where *γ*_*T*_, *γ*_*M*_, and *γ*_*K*_ represent the difference in allele frequency in the two populations at crossing for loci *T/t*, *M/m*, and *K/k*, respectively. A full derivation of eq. (5) is given in the Additional file 1: Appendix 1.

### Break-up of third order linkage disequilibrium by recombination

Third order linkage disequilibrium generated by admixture of populations is eroded by repeated recombination events under random mating over multiple generations. The break-up of high order linkage disequilibrium depends on the recombination fraction between the markers as it does for second order linkage disequilibrium. Hill [14, 15] has provided a full account of the decline of high order linkage disequilibria up to six loci. He showed that the three-locus disequilibrium declines exponentially at all generations. In order to compare our observed genome-wide results of third order disequilibrium, we have computed the break-up of third order linkage disequilibrium with recombination. The haplotype frequency at a generation *t*, is given by:

$$ {f}_{ij k}^t=\left(1-{c}_{T\mathrm{M}}\right)\left(1-{c}_{MK}\right){f}_{ij k}^{t-1}+{c}_{TM}\left(1-{c}_{MK}\right){f}_{i..}^{t-1}{f}_{. jk}^{t-1}+{c}_{MK}\left(1-{c}_{TM}\right){f}_{ij.}^{t-1}{f}_{..k}^{t-1}+{c}_{MK}{c}_{TM}{f}_{i..}^{t-1}{f}_{.j.}^{t-1}{f}_{..k}^{t-1}, $$

where *c*_*TM*_ and *c*_*MK*_ are the recombination fractions between *T/t* and *M/m*, and between *M/m* and *K/k*, respectively; ${f}_{ijk}^{t-1}$ represents the frequency of *ijk* in generation *t*-1. Dots in the subscripts of this equation are used to represent either allele (two dots) or two-locus haplotype (one dot) frequencies corresponding to haplotype *ijk*. For exmple *f*_.jk_ is the frequency of haplotype with alleles* jk*  at the last two loci, *M/m* and *K/k*. This model assumes no interference.

Third order linkage disequilibrium can be obtained in each generation, *t*, by making use of the dynamics of haplotype and allele frequencies. For example, for *ijk = TMK* the disequilibrium is

$$ {\delta}_{TM K}^t={f}_{TM K}^t-{f}_T^t{f}_M^t{f}_K^t-{f}_T^t{\delta}_{MK}^t-{f}_M^t{\delta}_{TK}^t-{f}_K^t{f\delta}_{TM}^t. $$

The maximum third order linkage disequilibrium occurs when the three loci are at intermediate allele frequencies, and second order disequilibria are zero (*δ*_*TM*_ = *δ*_*TK*_ = *δ*_*MK*_ = 0). In this situation, *δ*_*TMK*_ must range between $-\frac{1}{8}$ and $\frac{1}{8}$, which represents the limits of this parameter. If ${\delta}_{TMK}=\frac{1}{8}$ then only four haplotypes are segregating (*TMK*, *tmK*, *Tmk*, *tMk*), none of them with alleles complementary to each other. We investigated the break-up of linkage disequilibrium in this situation, in which third order linkage disequilibrium is the highest possible. The break-up of third order linkage disequilibrium was computed with the equation of third order linkage disequilibrium (${\delta}_{TMK}^t$) after substituting haplotype frequencies ${f}_{ijk}^t$.

### Normalization of the third order linkage disequilibrium parameter

The values of second and higher order linkage disequilibrium coefficients depend on allele frequencies and on lower order disequilibria coefficients, making them difficult to interpret since the same estimates of linkage disequilibrium maybe high when allele frequencies are near 0 or 1 but low when frequencies are intermediate. In order to partially mitigate this limitation, Lewontin in 1964 proposed *D’* as a measure for normalizing second order linkage disequilibrium [16]. This method forces the second order linkage disequilibrium parameter to be between − 1 and + 1, which basically provides information on the disequilibrium given the allele frequencies. An extension of the normalization to the third order linkage disequilibrium parameter has been proposed by Thomson and Baur [7]. Latter on, Robinson et al. [10] defined normalized third order linkage disequilibrium “as the amount by which the observed disequilibrium values exceed the minimum possible of that sign, divided by the length of the total range of admissible disequilibria with the observed sign”. The normalization of third order linkage disequilibrium serves the same purpose as the normalization of second order linkage disequilibrium, i.e., to provide a relative measure of the importance of the disequilibrium for the given allele frequencies. The normalization of third order linkage disequilibrium is much more complicated because the second order coefficients impose restrictions on the values of third order linkage disequilibrium. After we used Robinson et al. [10] method to normalized third order linkage disequilibrium in our data we observed that the great majority of estimates were 1 or − 1 or undefined in which *δ*_*TMK*_ can only take one possible value given allele frequencies and pairwise linkage disequilibrium parameters [10]. It is, therefore, not very useful when describing the extension of third order linkage disequilibrium in animal or plant populations. We propose an alternative measure of the relative value of third order disequilibrium versus second order disequilibrium by

$$ {\beta}_{TM K}=\frac{abs\left({\delta}_{TM K}\right)}{abs\left({\delta}_{TM K}\right)+ abs\left({\delta}_{TM}\right)+ abs\left({\delta}_{MK}\right)+ abs\left({\delta}_{TK}\right)} $$

This equation just provides information on how important is third order linkage disequilibrium relative to second order linkage disequilibrium in the context where mutual constrains exist between these coefficients. This parameter has values between 0 and 1, with 1 meaning that all disequilibrium is third order, and with 0 that all disequilibrium is second order. Values over 0.5 indicate that most of the disequilibrium is of third order.

### A computer algorithm for the estimation of third order linkage disequilibrium

Consider again three SNPs, *T/t*, *M/m*, and *K/k*. It is assumed that the population is in Hardy-Weinberg equilibrium. Following Geiringer [5], Bennet [6] and Thompson and Baur [7], the haplotype frequencies as a function of allele frequencies (*f*_*T*_, *f*_*M*_, *f*_*K*_), second order (*δ*_*TM*_, *δ*_*MK*_, *δ*_*TK*_), and third order linkage disequilibrium parameters (*δ*_*TMK*_) are:

6 $$ {\displaystyle \begin{array}{l}{f}_{TM K}={f}_T{f}_M{f}_K+{f}_T{\delta}_{MK}+{f}_M{\delta}_{TK}+{f}_K{\delta}_{TM}+{\delta}_{TM K}\\ {}{f}_{TmK}={f}_T{f}_m{f}_K-{f}_T{\delta}_{MK}+{f}_m{\delta}_{TK}-{f}_K{\delta}_{TM}-{\delta}_{TM K}\\ {}{f}_{tMK}={f}_t{f}_M{f}_K+{f}_t{\delta}_{MK}-{f}_M{\delta}_{TK}-{f}_K{\delta}_{TM}-{\delta}_{TM K}\\ {}{f}_{tmK}={f}_t{f}_m{f}_K-{f}_t{\delta}_{MK}-{f}_m{\delta}_{TK}+{f}_K{\delta}_{TM}+{\delta}_{TM K}\\ {}{f}_{TM k}={f}_T{f}_M{f}_k-{f}_T{\delta}_{MK}-{f}_M{\delta}_{TK}+{f}_k{\delta}_{TM}-{\delta}_{TM K}\\ {}{f}_{Tmk}={f}_T{f}_m{f}_k+{f}_T{\delta}_{MK}-{f}_m{\delta}_{TK}-{f}_k{\delta}_{TM+}{\delta}_{TM K}\\ {}{f}_{tMk}={f}_t{f}_M{f}_k-{f}_t{\delta}_{MK}+{f}_M{\delta}_{TK}-{f}_k{\delta}_{TM}+{\delta}_{TM K}\\ {}{f}_{tmk}={f}_t{f}_m{f}_k+{f}_t{\delta}_{MK}+{f}_m{\delta}_{TK}+{f}_k{\delta}_{TM}-{\delta}_{TM K}\end{array}} $$

To better understand the range of possible values and the relationship of third order linkage disequilibrium with second order parameters, the following particular cases were investigated:

- Intermediate allele frequencies at the three loci (*f*_*T*_ *= f*_*M*_ *= f*_*K*_ = 0.5) and zero second order disequilibria between all pairs (*δ*_*TM*_ = *δ*_*TK*_ = *δ*_*MK*_ = 0). After using equation (6), the haplotype frequencies are: ${f}_{TMK}=\frac{1}{8}+{\delta}_{TMK}$, ${f}_{TmK}=\frac{1}{8}-{\delta}_{TMK}$, ${f}_{tMK}=\frac{1}{8}-{\delta}_{TMK}$, ${f}_{tmK}=\frac{1}{8}+{\delta}_{TMK}$, ${f}_{TMk}=\frac{1}{8}-{\delta}_{TMK}$, ${f}_{Tmk}=\frac{1}{8}+{\delta}_{TMK}$,${f}_{tMk}=\frac{1}{8}+{\delta}_{TMK}$, and ${f}_{tmk}=\frac{1}{8}-{\delta}_{TMK}$. Consequently, *δ*_*TMK*_ must range between $-\frac{1}{8}$ and $\frac{1}{8}$, because all haplotype frequencies must be zero or positive. If ${\delta}_{TMK}=\frac{1}{8}$ then ${f}_{TMK}={f}_{tmK}={f}_{Tmk}={f}_{tMk}=\frac{1}{4}\kern0.5em$, and *f*_*Tmk*_ = *f*_*tMK*_ = *f*_*TMk*_ = *f*_*tmk*_ = 0. Then, only four haplotypes are segregating (*TMK*, *tmK*, *Tmk*, *tMk*), none of them with complementary alleles to each other.
- Intermediate allele frequencies for the three loci (*f*_*T*_ *= f*_*M*_ *= f*_*K*_ = 0.5), and maximum second order LD (*δ*_*TM*_ = *δ*_*TK*_ = *δ*_*MK*_ = 0.25). Then, the haplotype frequencies are ${f}_{TMK}=\frac{1}{2}+{\delta}_{TMK}$, *f*_*TmK*_ =  − *δ*_*TMK*_, *f*_*tMK*_ =  − *δ*_*TMK*_, *f*_*tmK*_ = *δ*_*TMK*_, *f*_*TMk*_ =  − *δ*_*TMK*_, *f*_*Tmk*_ = *δ*_*TMK*_, *f*_*tMk*_ = *δ*_*TMK*_, and ${f}_{tMk}=\frac{1}{2}-{\delta}_{TMK}$. Consequently, *δ*_*TMK*_ = 0 and ${f}_{TMK}=\frac{1}{2}\kern0.5em$*,* ${f}_{tmk}=\frac{1}{2}\kern0.5em$ because all haplotype frequencies must be zero or positive.

Summarizing, the range of possible values of the third order linkage disequilibrium are between − 0.125 and + 0.125 and equal to zero when second order linkage disequilibria are at their maximum values. Only haplotypes *TMK*, *tmK*, *Tmk*, and *tMk* will be segregating for full third order linkage disequilibrium (*δ*_*TMK*_ = 1/8) at intermediate allele frequencies and in absence of any second order linkage disequilibrium. That is, allele *K* will be associated to haplotypes *TM* and *tm*, and allele *k* to haplotypes *Tm* and *tM.* A similar argument can be done for *δ*_*TMK*_ = − 1/8 with allele *k* is associated to haplotypes *TM* and *tm*, and allele *K* to haplotypes *Tm* and *tM.*

The likelihood to estimate linkage disequilibrium parameters and allele frequencies for three loci when using genotypic information data from diploid individuals is:

7 $$ L\left({\delta}_{TM K},{\delta}_{TM},{\delta}_{TK},{\delta}_{MK},{f}_T,{f}_M,{f}_K| NG\right)=K{\prod}_{i=1}^{27}{\varphi_i^n}^i $$

where *K* is a constant, *NG* is the triplet genotypic information on diploid individuals, ${\varphi}_i$ is the probability (or frequency) of the observed *i-th* triplet genotype, and *n*^*i*^ is the number of individuals (counts) having the *i-th* triple genotype in the population. By triple genotype, we mean the joint genotype at the three loci *T/t*, *M/m*, and *K/k*. The 27 triplet genotype probabilities, *φ*_*i*_ can be obtained from Table 2. For example, the probability of observing a triple heterozygote, *TtMmKk*, is *φ*_*TtMmKk*_ *= 2(f*_*TMK*_*f*_*tmk*_ *+ f*_*tMk*_*f*_*TmK*_*+ f*_*Tmk*_*f*_*tMK*_*+ f*_*TMk*_*f*_*tmK*_*)* and the corresponding number of observed triple heterozygotes is *n*^*TtMmKk*^.

**Table 2:** Frequencies of sire and dam gametes for all possible combinations of alleles at three SNPs (with alleles T/t M/m and K/k) to produce the 27 genotypes

	Sire	TMK	TmK	tMK	tmK	TMk	Tmk	tMk	tmk
Dam	Freq	*f*_TMK_	*f*_TmK_	*f*_tMK_	*f*_tmK_	*f*_TMk_	*f*_Tmk_	*f*_tMk_	*f*_tmk_
TMK	*f*_TMK_	TTMMKK *f*_TMK_* f*_TMK_	TTMmKK *f*_TMK_ * f*_TmK_	TtMMKK *f*_TMK_ *f*_tMK_	TtMmKK *f*_TMK_*f*_tmK_	TTMMKk *f*_TMK_*f*_TMk_	TTMmKk *f*_TMK_*f*_Tmk_	TtMMKk *f*_TMK_* f*_tMk_	TtMmKk *f*_TMK_*f*_tmk_
TmK	*f*_TmK_	TTMmKK *f*_TmK_ f_TMK_	TTmmKK *f*_TmK_ *f*_TmK_	TtMmKK *f*_TmK_*f*_tMK_	TtmmKK *f*_TmK_*f*_tmK_	TTMmKk *f*_TmK_*f*_TMk_	TTmmKk *f*_TmK_*f*_Tmk_	TtMmKk *f*_TmK_*f*_tMk_	TtmmKk *f*_TmK_*f*_tmk_
tMK	*f*_tMK_	TtMMKK *f*_tMK_* f*_TMK_	TtMmKK *f*_tMK_*f*_TmK_	ttMMKK *f*_tMK_* f*_tMK_	ttMmKK *f*_tMK_*f*_tmK_	TtMMKk *f*_tMK_*f*_TMk_	TtMmKk *f*_tMK_*f*_Tmk_	ttMMKk *f*_tMK_*f*_tMk_	ttMmKk *f*_tMK_ *f*_tmk_
tmK	*f*_tmK_	TtMmKK *f*_tmK_ f_TMK_	TtmmKK *f*_tmK_*f*_TmK_	ttMmKK *f*_tmK_*f*_tMK_	ttmmKK *f*_tmK_*f*_tmK_	TtMmKk *f*_tmK_*f*_TMk_	TtmmKk *f*_tmK_*f*_Tmk_	ttMmKk *f*_tmK_*f*_tMk_	ttmmKk *f*_tmK_*f*_tmk_
TMk	*f*_TMk_	TTMMKk f_TMk_ f_TMK_	TTMmKk *f*_TMk_*f*_TmK_	TtMMKk *f*_TMk_*f*_tMK_	TtMmKk *f*_TMk_*f*_tmK_	TTMMkk *f*_TMk_*f*_TMk_	TTMmkk *f*_TMk_*f*_Tmk_	TtMMkk *f*_TMk_*f*_tMk_	TtMmkk *f*_TMk_*f*_tmk_
Tmk	*f*_Tmk_	TTMmKk f_Tmk_ f_TMK_	TTmmkK *f*_Tmk_*f*_TmK_	TtMmKk *f*_Tmk_*f*_tMK_	TtmmKk *f*_Tmk_*f*_tmK_	TTMmkk *f*_Tmk_*f*_TMk_	TTmmkk *f*_Tmk_* f*_Tmk_	TtMmkk *f*_Tmk_*f*_tMk_	Ttmmkk *f*_Tmk_*f*_tmk_
tMk	*f*_tMk_	TtMMKk f_tMk_ f_TMK_	TtMmKk *f*_tMk_*f*_TmK_	ttMMKk *f*_tMk_*f*_tMK_	ttMmKk *f*_tMk_*f*_tmK_	TtMMkk *f*_tMk_*f*_TMk_	TtMmkk *f*_tMk_*f*_Tmk_	ttMMkk *f*_tMk_*f*_tMk_	ttMmkk *f*_tMk_ *f*_tmk_
tmk	*f*_tmk_	TtMmKk f_tmk_ f_TMK_	TtmmKk *f*_tmk_*f*_TmK_	ttMmKk *f*_tmk_*f*_tMK_	ttmmKk *f*_tmk_*f*_tmK_	TtMmkk *f*_tmk_*f*_TMk_	Ttmmkk *f*_tmk_*f*_Tmk_	ttMmkk *f*_tmk_*f*_tMk_	ttmmkk *f*_tmk_ *f*_tmk_

The likelihood equation is maximized for a third order linkage disequilibrium parameter, three second order linkage disequilibrium parameters, and three allele frequencies corresponding to each of the three SNPs. Therefore, the model has seven degrees of freedom corresponding to the eight haplotypes minus one. Solving equation (7) is not trivial because of the constraints imposed by second order linkage disequilibrium parameters and allele frequencies. It means that the parameter space for each of the parameters to be estimated (third and second order linkage disequilibrium coefficients and allele frequencies) depends on the values of the other parameters. The EM algorithm is an iterative procedure in which successive sets of haplotype frequencies are computed, starting with initial arbitrary values. In the expectation step, haplotype frequencies are used as if they were the unknown true frequencies to estimate genotype frequencies. In the maximization step, these expected genotype frequencies are used in turn to estimate haplotype frequencies at the next iteration. The process is repeated until haplotype frequencies in consecutive iterations are less than some small value, i.e., until convergence is reached. For multi-allelic loci, Long et al.*.*. [9] pointed out two methods after implementing an EM algorithm: 1) computing one by one the probabilities corresponding to each of the 27 triplet genotypes (Table 2), and 2) generating all haplotypes that could form a genotype that are compatible with each triple genotype. The first method, according to Long et al. [9], requires a large amount of program code and is impractical for more than three loci. The second method is computer intensive because it also requires the generation and evaluation of many genotypes that could not have produced the triplet genotype. In line with the second method of Long et al [9] and just for bi-allelic loci, we have developed and implemented an EM algorithm in a subroutine that is simple, fast, and straightforward to extend for the estimation of linkage disequilibrium to more than three biallelic loci. The eight haplotype frequencies are stored in an array with three dimensions (three loci), with allele values of “1” or “2” in each dimension corresponding to alleles “1” or “2”. The steps for the E-M algorithm are:

I) Set initial haplotype frequencies (arbitrarily),
II) Expectation step in which genotype frequencies are estimated based on haplotype frequencies from step I. In order to resolve to which haplotypes may correspond observed genotype counts the proportion of double or triple heterozygotes in coupling or repulsion for each haplotype needs to be computed. In programing in Fortran we used code 3 for individuals with the heterozygote genotype. For example, for haplotype “111”, the proportion of individuals with genotype heterozygote at the two first loci and homozygote with allele 1 at the third loci is:

$$ {W}_{331}=\frac{f_{111}{f}_{221}}{f_{111}{f}_{221}+{f}_{121}{f}_{211}} $$

Similarly, the proportion for homozygote (for allele 1) individuals at the first locus and heterozygote at the last two loci is:

$$ {W}_{133}=\frac{f_{111}{f}_{122}}{f_{111}{f}_{122}+{f}_{112}{f}_{121}} $$

In the same way, the proportion of individuals with other genotypes are:

$$ {W}_{313}=\frac{f_{111}{f}_{212}}{f_{111}{f}_{212}+{f}_{211}{f}_{112}} $$

$$ {W}_{333}=\frac{f_{111}{f}_{222}}{f_{111}{f}_{222}+{f}_{221}{f}_{112}+{f}_{211}{f}_{122}+{f}_{212}{f}_{121}} $$

The products between frequencies of haplotypes in the denominator correspond to all possible combinations of complementary haplotypes that could result in genotype as the subscript of *W*. This process is done for the eight haplotype frequencies.

III) The maximization step consists in estimating haplotype frequencies using genotype counts observed or as estimated in step II. For example for the haplotype “111”, the frequency to be estimated is:

$$ {f}_{111}^{\ast }=\left(1/2N\right)\left(2{w}^{111}+{w}^{311}+{w}^{131}+{w}^{113}+{W}_{331}{w}^{331}+{W}_{313}{w}^{313}+{W}_{133}{w}^{133}+{W}_{333}{w}^{333}\right) $$

where *w*^*ijk*^ represents the counts for genotype *ijk* with values 1 or 2 for alleles 1 or 2, and 3 for the heterozygote. All other seven haplotypes are constructed in the same way.

IV) Go to step II until convergence of haplotype frequencies is reached.

This algorithm is simple and suitable for fast computing when implemented in a computer language such as Fortran90. In this implementation, the number of individuals for each triple genotype is stored in an array with three dimensions. Each one corresponds to one locus and there are three alternatives: “1” and “2” are used for homozygotes, and “3” for heterozygotes. Source code for estimating haplotype frequencies in Fortran90 is provided in the Additional file 2: Appendix 2. Linkage disequilibrium parameters can be easily estimated from haplotype frequencies using equation (6).

### Analysis of third order linkage disequilibrium in IBERIAN pigs

#### Animal material

Genotypes from 306 sows belonging to a composite line (Torbiscal) genetically isolated between 1963 and 2013 and resulting from the blending of four ancient Spanish and Portuguese Iberian breed strains [17], were used in this study.

### Genotyping of SNPs

DNA samples were isolated from blood using a standard phenol/chloroform protocol and genotyped with the Illumina Porcine SNP60 BeadChip [18] and the Infinium HD Assay Ultra protocol (Illumina Inc.). Genotypes of 62,163 SNP probes were analyzed with the Genome Studio software (Illumina). Data quality control was performed according to the following criteria: call rate of the sample > 0.96; SNPs with a call rate > 0.99; GenTrain Score > 0.70; AB R Mean > 0.35; and MAF > 0.05. SNPs located on sex chromosomes, those not mapped in the Sscrofa10.2 assembly, or those with inconsistent inheritance from dam to daughter were also removed. Only 26,347 polymorphic SNPs were retained and used for further analyses.

#### Inter and intra-chromosomal third order linkage disequilibrium

Estimation of the third order linkage disequilibrium parameter was carried out for all triplets of three consecutive SNPs for each of the autosomal chromosomes. This will be referred to as short range intra-chromosomal third order linkage disequilibrium. The total number of triplets was 26,311. In addition, inter-chromosomal third order linkage disequilibrium was estimated by randomly drawing three out of the 18 autosomal chromosomes and by selecting randomly one SNP within each chromosome. In order to make an easier comparison between inter and intra-chromosomal third order linkage disequilibrium, the process was repeated 26,311 times, the same number as for the intra-chromosomal third order linkage disequilibrium. Similar to the second order linkage disequilibrium, the third order linkage disequilibrium is expected to be negligible in most inter-chromosomal situations.

#### Third order linkage disequilibrium and physical distance

An extensive analysis in the smaller chromosome (SSC12) was carried out in order to investigate the relationship between third order linkage disequilibrium and physical distance. Third order linkage disequilibria were estimated among all possible triplets in a rolling chromosomal fragment of 400 SNPs. The total number of analyses was 35,784,200. In addition, the relationship of the proposed normalized third order linkage disequilibrium *β*_*TMK*_, with the number of haplotypes and a likelihood ratio test was investigated. The likelihood ratio test was computed using the full model, i.e., estimating all parameters versus a model with only allele frequencies (model M15 of Long et al. [9]).

## Results

The range of possible values of the third order linkage disequilibrium is between − 0.125 and + 0.125 and is equal to zero when second order linkage disequilibria are at their maximum values. Table 3 shows some examples on how third order linkage disequilibrium can be generated by population admixture after using equation (5). Second order coefficients were chosen with similar or distant values in order to understand the amount of third order linkage disequilibrium generated. It is not only necessary that the loci differ in allele frequency but also in the pairwise linkage disequilibrium. The larger the differences in allele frequency, the larger the third order linkage disequilibrium generated. If allele frequencies and pairwise linkage disequilibrium coefficients are the same in the two populations at crossing then third order linkage disequilibrium is not generated and it is just the average of the third order disequilibrium parameter of the two populations at crossing.

**Table 3:** Third order linkage disequilibrium generated in population Z (${\delta}_{TMK}^Z$) after admixture of populations X and Y with alternative allele frequencies and second order linkage disequilibrium parameters. It assumes *τ*=0.5 and ${\delta}_{TMK}^X=0$, ${\delta}_{TMK}^Y=0$

${f}_T^X$	${f}_M^X$	${f}_K^X$	${f}_T^Y$	${f}_M^Y$	${f}_K^Y$	${\delta}_{MK}^X$	${\delta}_{TK}^X$	${\delta}_{TM}^X$	${\delta}_{MK}^Y$	${\delta}_{TK}^Y$	${\delta}_{TM}^Y$	${\delta}_{TMK}^Z$
0.8	0.8	0.8	0.2	0.2	0.2	0	0	0	0	0	0	0
0.8	0.2	0.8	0.2	0.8	0.2	0	0	0	0	0	0	0
0.5	0.5	0.5	0.5	0.5	0.5	0.25	0.25	0.25	0	0	0	0
0.5	0.5	0.5	0.5	0.5	0.5	0.25	0.25	0	0	0	0	0
0.5	0.5	0.5	0.5	0.5	0.5	0.25	0	0	0	0	0	0
0.5	0.5	0.5	0.2	0.2	0.2	0.25	0.25	0.25	0	0	0	0.056
0.5	0.5	0.5	0.2	0.2	0.2	0.25	0.25	0	0	0	0	0.038
0.5	0.5	0.5	0.1	0.1	0.1	0.25	0.25	0.25	0	0	0	0.075
0.5	0.5	0.5	0.01	0.01	0.01	0.25	0.25	0.25	0	0	0	0.092

**Fig. 2:**
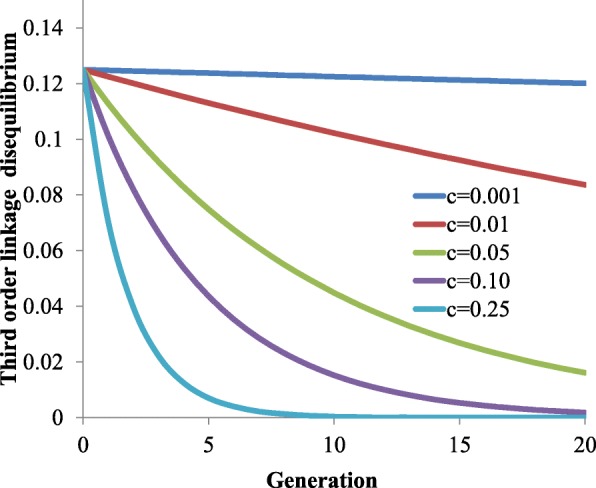
Break-up of third order linkage disequilibrium over time due to recombination. The same recombination fraction between each pair of consecutive SNPs, *c*, in a triplet is assumed

Once third order linkage disequilibrium is generated by population admixture, selection or genetic drift, its value is eroded by recombination. Figure 2 shows an example in which population admixture generated the maximum possible value of third order linkage disequilibrium to be eroded by recombination for alternative values of the recombination fraction. In this example, the same recombination fraction (*c*) is assumed between every two consecutive markers. The break-up of third order linkage disequilibrium is quick at a high recombination fraction but much lower for markers with low recombination fraction. It may take many generations for the LD to be fully eroded. For example, for *c* = 0.001 (~100kb), third order linkage disequilibrium reduces only marginally in 20 generations.

**Fig. 3:**
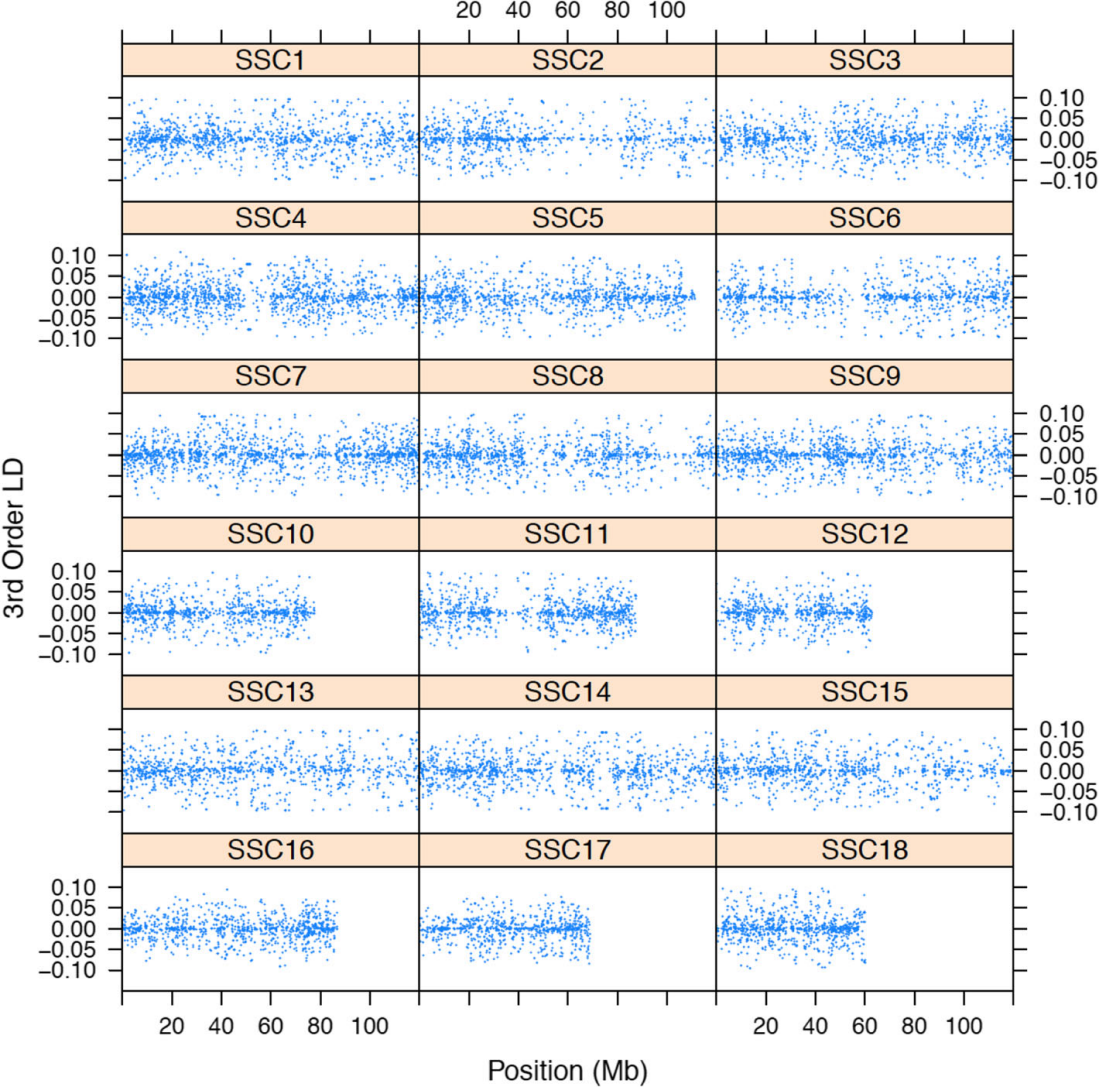
Third order linkage disequilibrium parameter estimates for the 18 autosomes of a closed line of Iberian pigs

Figure 3 shows the third order linkage disequilibrium estimates for consecutive SNPs along their position for the 18 autosomal chromosomes. The average distance across the genome between the two more extreme SNPs in a triplet was 185.2 kb (SD = 214.1). The third order linkage disequilibrium parameter can take any value within its range although zero is not uncommon (3). The correlation between the absolute value of the third order linkage disequilibrium parameter and the distance between the two more extreme SNPs was − 0.115 (SE = 0.006), indicating that the larger the distance, the lower the third order disequilibrium. This computation only used adjacent markers, which are located close to each other. The correlation between the average distance between the three markers and the third order linkage disequilibrium coefficient for over 35 million triplets in SSC12 (allowing longer distances between markers) was − 0.23 (SE = 0.00016).

**Fig. 4:**
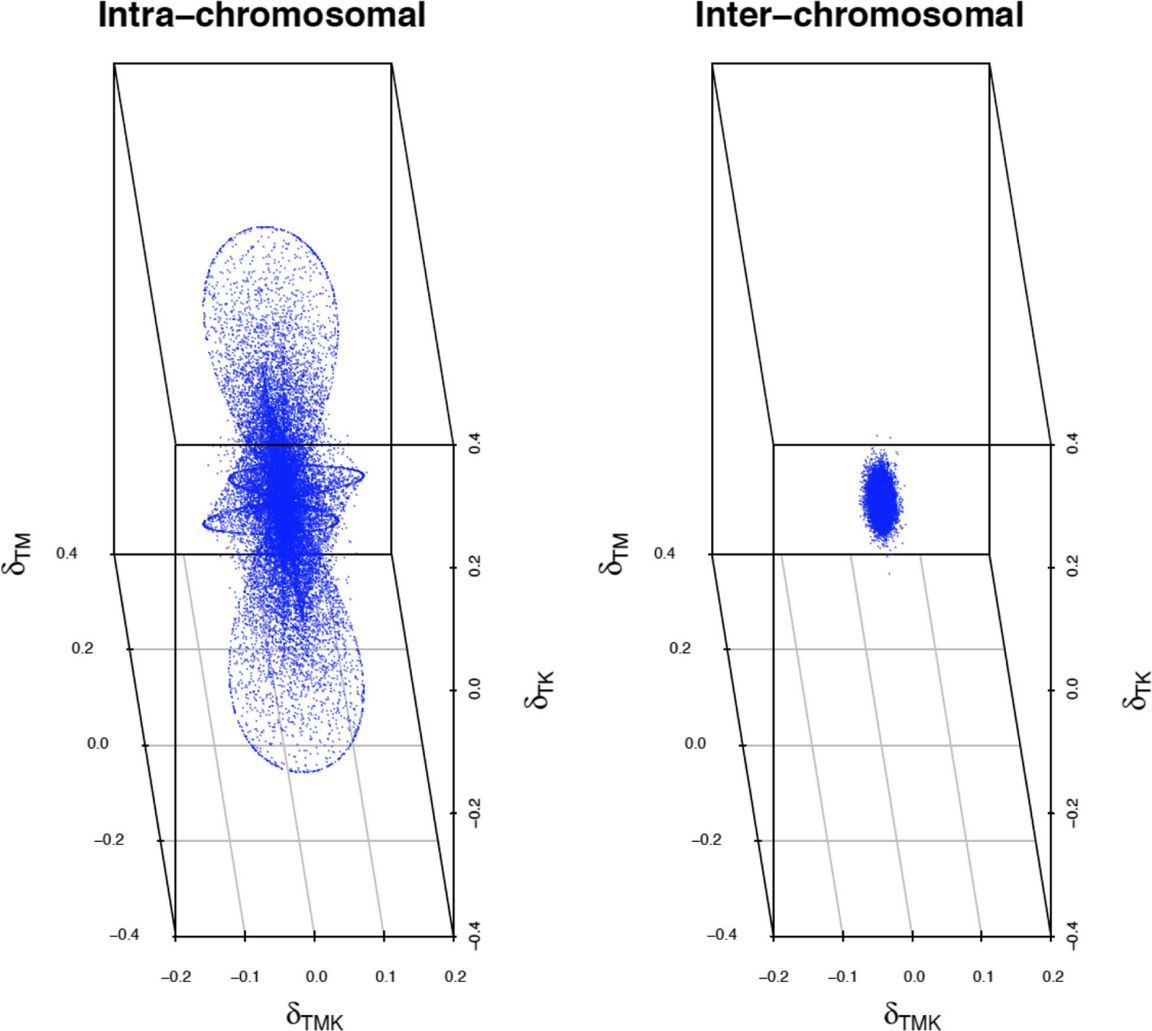
Three dimension plot of two of the estimates of the second order linkage disequilibrium parameter (*δ*_*TM*_*,* and *δ*_*TK*_) versus estimates of the third order linkage disequilibrium parameter (*δ*_*TMK*_) for all consecutive SNP triplets (Inter-chromosomal) and random SNPs from three different chromosomes (Inter-chromosomal)

**Fig. 5:**
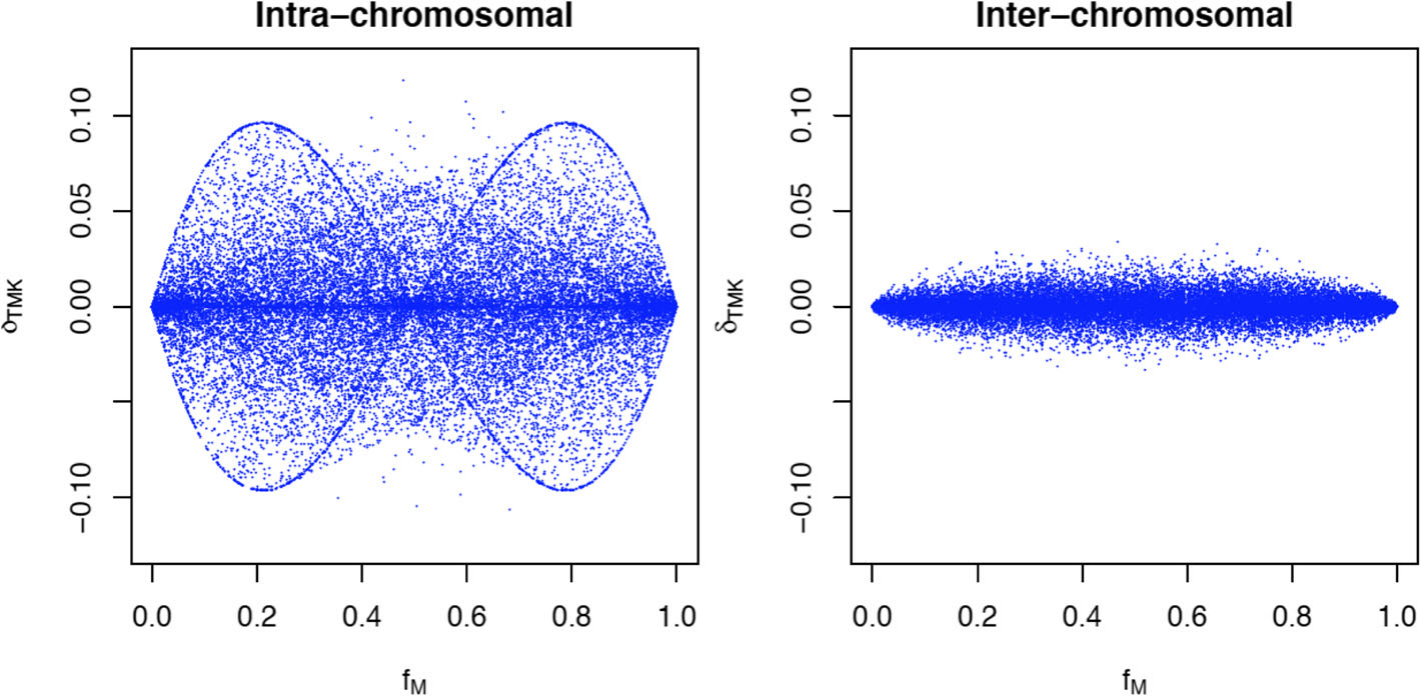
Plot of the third order linkage disequilibrium parameter (*δ*_*TMK*_) versus one of the allele frequencies in consecutives (intra-chromosomal) and random triplets (inter-chromosomal)

**Fig. 6:**
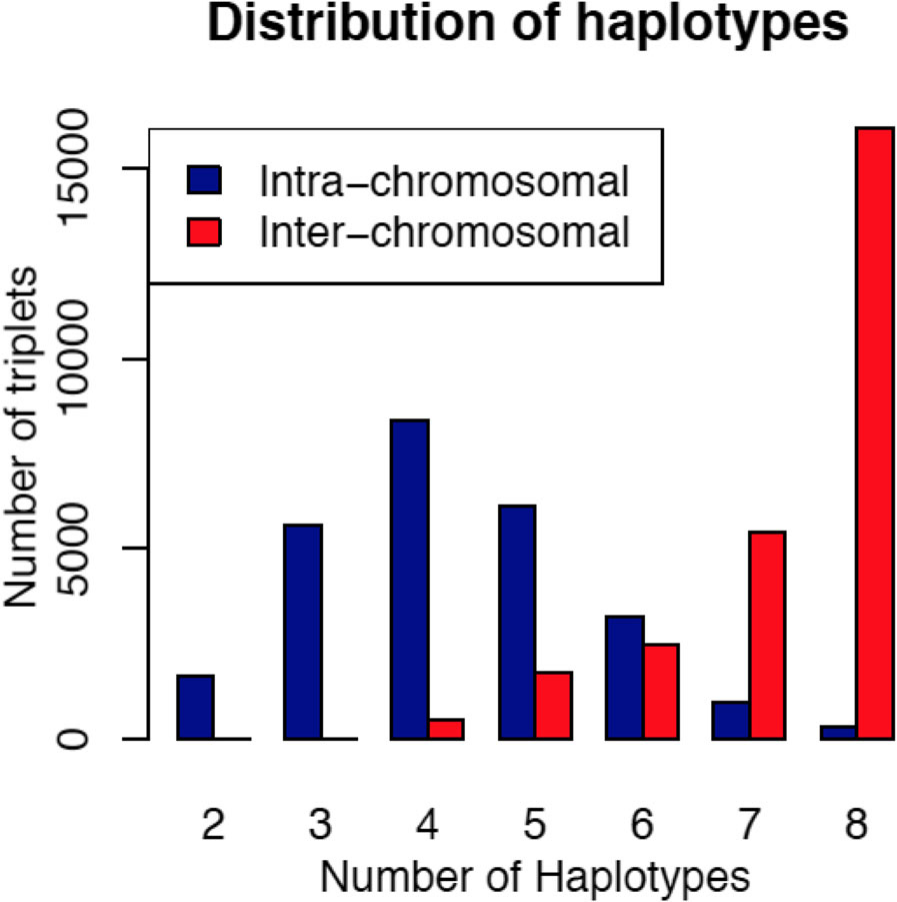
Histogram of frequencies of the observed distribution of the number of haplotypes in intra-chromosomal (blue) and inter-chromosomal (red)

Plots of both inter- and intra-chromosomal third order linkage disequilibria versus two of the second order linkage disequilibria parameters illustrate the relationship of second and third order disequilibria (Fig. 4). There is two and three locus disequilibrium when all loci are closely linked. Inter-chromosomal disequilibria are generally around zero for both second and third order linkage disequilibria. Figure 4 also illustrates that these parameters impose constraints to each other. Figure 5 shows the relationships between the third order disequilibrium and the allele frequency at one of the SNPs. The allele frequencies at the two other markers ranged from 0 to 1 since these represent estimates obtained from the data. Although the expected maximum third order linkage disequilibrium is 0.125 (absolute value), it was observed that values were near 0.09 when allele frequencies were 0.2 or 0.8, likely due to constraints imposed by allele frequencies at the other SNPs and second order linkage disequilibrium coefficients. Figure 6 shows the histogram of the number of haplotypes for both intra and inter-chromosomal analyses. The average number of haplotypes was 4.3 and 7.3 for intra and inter-chromosomal analyses, respectively. The number of haplotypes was much lower when considering triplets of consecutive SNPs in the same chromosome because of the reduced chance of recombination between them.

Following Robinson et al. (1991), the results of the estimates of normalized third order linkage disequilibrium were 0 (14.9%), ± 1 (20%), or an undefined situation in which the third order disequilibrium parameter is restricted to one single value given allele frequencies and second order disequilibrium parameters (61.5%). Only 3.6% of all the analyses resulted in a third order parameter different from 0, 1, − 1 or the undefined situation. This may be related to the number of haplotypes (and/or constraints imposed by allele frequencies and second order linkage disequilibria) because there was not a single estimate of undefined situation, 1, − 1 or 0 among the 309 triplets with eight haplotypes. There were only seven out of 1648 triplets for which estimates of normalized third order linkage disequilibrium were different from undefined situation, 1, − 1 or 0 among triplets with just two haplotypes. Therefore, normalizing third order linkage disequilibrium as proposed by Robinson et al., (1991) is not useful for deciphering situations involving closely linked markers.

**Fig. 7:**
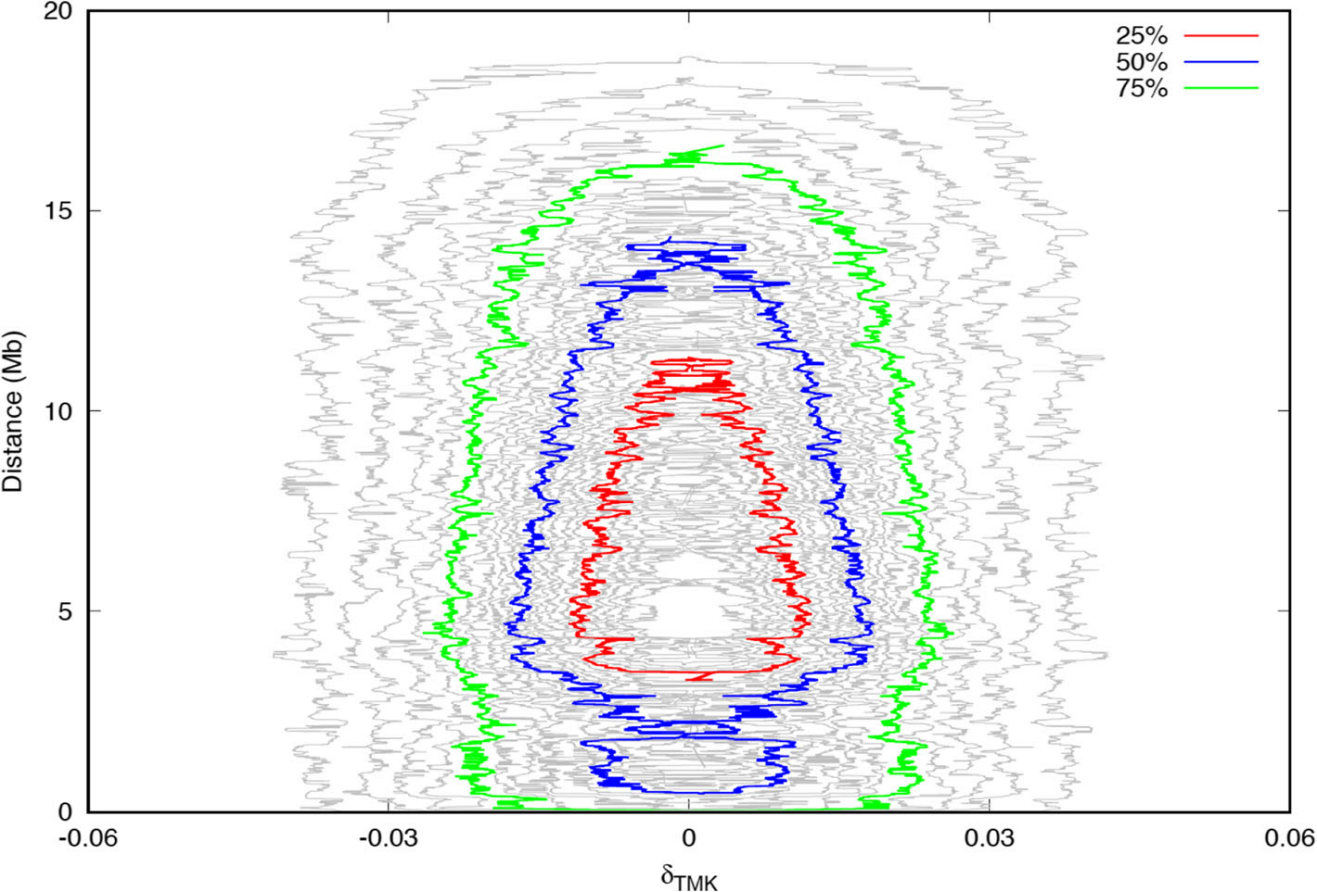
Plot of the third order linkage disequilibrium versus physical distance in over 35 millions triplets of SSC12. Red, blue and green lines represent the 25, 50 and 75% percentiles of the distribution. There are four intervals of one percentile each between each of the two: 25, 50 and 75% percentiles

Figure 7 illustrates the relationship of third order linkage disequilibrium with physical distance on SSC12. This figure provides 25th percentile, 50th percentile, and 75th percentile in the analysis of third order linkage disequilibrium over 35 million triplets. There was not a high proportion of triplets with a high value of disequilibrium for distances of less than 3.5 to 4 Mb. A larger proportion of triplets (with values of third order linkage disequilibrium up to ±0.015) was found at 4 Mb. Then, the proportion of triplets with third order linkage disequilibrium declines near to zero for distances over 15 Mb.

**Fig. 8:**
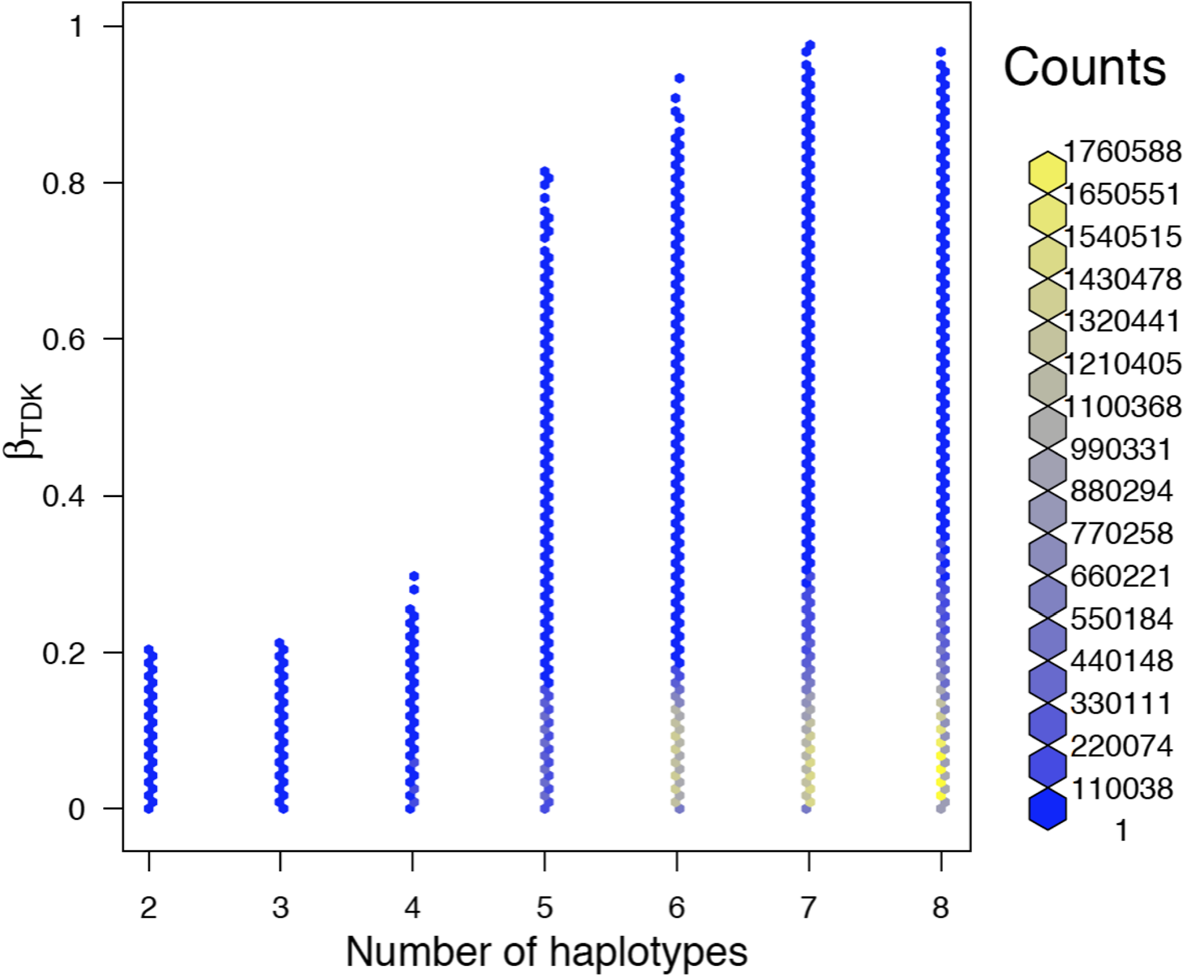
Hexbin histogram of the distribution of the number of haplotypes in a triplet with the proportion of third order linkage disequilibrium versus all disequilibria *(β*_*TMK*_) distance in over 35 millions triplets of SSC12. The plot was carried out with hexbin package of R, which is a set of functions for creating, manipulating and plotting hexagon bins. The colors on the right hand side of the figure illustrate the amount of counts (associate to each color) that are represented for each hexagon in the figure

In addition, the use of the newly proposed measure to estimate the proportion of third order linkage disequilibrium revealed that a high proportion of third order linkage disequilibrium versus all disequilibria seems to be associated to a larger number of haplotypes (Fig. 8).

## Discussion

**Fig. 9:**
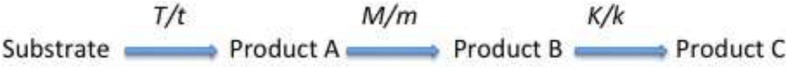
Fictive metabolic pathway illustrating how third order linkage disequilibrium could lead to epistasis

The importance of gene interactions in the expression of phenotypic traits is gaining interest in the scientific community [19]. The biological role of third order linkage disequilibrium can only be associated to epistasis in which combinations of alleles at different loci may trigger alternative metabolic pathways. The putative role of third order linkage disequilibrium is not general to all possible models of gene interactions or epistasis. As we showed earlier, the association of one allele to more than one complementary haplotypes (not alleles at individual loci) is necessary for third order linkage disequilibrium to exist. For example, allele *K* must be associated to haplotypes *TM* and *tm*, and allele *k* to haplotypes *Tm* and *tM.* A fictive metabolic pathway that could represent this situation is represented in Fig. 9. Similar epistatic models have been previously investigated [20]. Alleles T and *M* increase products A and B, respectively. Alleles *t* and *m* reduce products A and B. Allele *K* at the third loci would only function for intermediate levels of product B to react to product C. Individuals with haplotypes *TM* would generate large amounts of product B. Individuals with haplotypes *tm* would generate small amounts of product B. Individuals with haplotypes *Tm or tM* would generate intermediate amounts of product B. Therefore, genetic systems with an excess of haplotypes TmK and/or tMK with respect to equilibrium would be selected when product C provides increased adaptation in a given environment. Thus, some metabolic pathways may trigger reactions for intermediate values of a product since merely second order linkage disequilibrium may not be enough to unravel the complexity of living organisms.

The results obtained in this study are based on the Expectation Maximization algorithm [8, 21], which assumes that the population is under random mating, and consequently, in Hardy-Weinberg equilibrium. The estimates of third order linkage disequilibrium may be affected by the fact that this population was created 50 years ago by the crossing of four strains of Iberian pigs. Our results, displayed graphically, support that third order linkage disequilibrium is not uncommon for linked loci in this composite line of Iberian pigs. In addition, the magnitude of the disequilibrium decreases with the distance between loci as supported by the negative correlation between the distance and the third order linkage disequilibrium (− 0.115). Although the magnitude of this correlation is not high, the correlation was calculated with triplets of adjacent SNPs in which the distances are small. By using over 35 million triplets on SSC12 but allowing for all possible distances between markers this correlation became − 0.23. In conclusion, third order linkage disequilibrium declines with distance between markers.

Normalization of third order linkage disequilibrium [10] for closely linked loci is not generally useful because a majority of the estimates were either − 1, 1 or undefined (only one possible value for a given allele and pairwise disequilibrium). This is attributable to the observed low number of haplotypes for consecutive SNPs, which ultimately originates from the joined forces of mutation and recombination. A low number of haplotypes reduces also the number of parameters to be estimated and may impose additional constraints on pairwise and third order linkage disequilibria. Under these circumstances, the value of third order linkage disequilibria should not be normalized, and estimates should be used directly but bearing in mind that their value is constrained by allele frequencies and pairwise linkage disequilibria. An alternative to normalizing is the use of the proportion of third order linkage disequilibria versus all disequilibria. It provides information on the value of the relative proportions of third and pairwise linkage disequilibria. This proportion appears to be related to the number of haplotypes. Triplets with much third order linkage disequilibrium tend to show a larger number of haplotypes. Additionally, they tend to have a lower likelihood ratio test. More research is needed to evaluate the usefulness of this parameter.

It has been proposed that the difference between normalized pairwise disequilibria estimated from analyses of third and second order disequilibria should shed light on hitchhiking selection in a method called “constrained disequilibrium values” [22, 23]. Robison et al., 1991 showed that a third locus imposes further bounds on second order linkage disequilibria coefficients. Differences in pairwise disequilibria, normalized in the two different ways, highlight the influence that a third locus may exert on the pairwise measure. These authors proposed that differences in the normalized measures could indicate which of the three loci has the selected mutant. This method could be applied to populations typed with SNP arrays. The problem is that, generally speaking, all typed SNPs are neutral and none of them could be assigned as a locus under selection. Also, the method of Robinson et al. [22] requires normalization of pairwise linkage disequilibrium with and without the constraint of a third locus. As discussed above, normalization of pairwise linkage disequilibrium may be also problematic for biallelic loci when constrained by a third locus.

If interested in testing the third order linkage disequilibrium at specific locations, one would carry out hypothesis testing by means of a likelihood ratio test within the maximum likelihood framework. This is not straightforward because the expected haplotype frequencies in the reduced model (M7 in Long et al. [9]) are not estimated directly using the maximum likelihood approach. Long et al. [9] suggested using an iterative proportional fitting approach [24] but this is not optimal in the sense that haplotype frequencies are forced to be between 0 and 1. This situation is much aggravated by the fact that in many cases the third order linkage disequilibrium parameter can just take one possible value when constrained by second order disequilibria and/or allele frequencies. Consequently, testing at specific locations whether the third order linkage disequilibrium is different from zero is challenging.

Third order linkage disequilibrium can be generated by population admixture. It follows a similar pattern as second order linkage disequilibrium. Needing, in addition to a difference in allele frequencies of the two populations at crossing, differences in their pairwise linkage disequilibrium parameters. The observed levels of third order linkage disequilibrium in the analyzed Torbiscal line led us to conclude that this disequilibrium might have been generated after the crossing of multiple strains of Iberian pigs some 50 years ago. Alternatively, genetic drift may have also had a role given the small population size of this strain. More research using other animal, plant or human populations with a different population history may help to understand if the levels of third order linkage disequilibrium are high, when relating to the crossing history of the populations in question.

Once population admixture has generated third order linkage disequilibrium, the three-locus disequilibrium declines exponentially over time by recombination [14]. However, it can persist for long periods of time if the distances are small and recombination infrequent. Our calculations for a recombination fraction of 0.001 (~ 100 kb) between each two consecutive SNPs in a triplet would allow the maintenance of third order linkage disequilibrium for long periods of time. The analyses of the Torbiscal data revealed an average of 92.6 kb for consecutive SNPs, and therefore, could explain well the observed levels of third order linkage disequilibrium in this strain.

The use of composite lines in pig breeding schemes is becoming quite popular for both sire and dam lines [25]. The levels of third order linkage disequilibria in these composite populations may be similar to those observed in our study or higher in the dam lines coming from Chinese-European origins, with larger differences in allele frequencies. More research is needed to understand its implications for gene mapping and/or as an aid to trace haplotypes to ancestor’s origins.

## Conclusions

The main conclusions of this paper are: a) the existence of third order linkage disequilibrium is substantial in a composite Iberian pig line, b) third order linkage disequilibrium in this strain might have been generated by admixture of four strains of Iberian pigs, c) the absolute value of the third order linkage disequilibrium decreases rapidly with a physical distance above 4 Mb, d) the number of haplotypes is much reduced for linked loci due to the mutual constraints of pairwise and third order linkage disequilibrium parameters, and e) normalization of third order disequilibria is not advised for closely linked biallelic loci. High order linkage disequilibrium might shed light on our understanding of the complex metabolic pathways in which multiple loci are involved. Much of the actual variation in quantitative traits might go unnoticed when analyzing just one or two loci at a time.

## Additional files

Additional file 1: **Appendix 1.** Derivation of third order disequilibrium in population admixture. (DOCX 19 kb)

Additional file 2: **Appendix 2.** Subroutine to estimate third order linkage disequilibrium. (DOCX 16 kb)
