# Supplementary material for: Extent of third-order linkage disequilibrium in a composite line of Iberian pigs
Source: BMC Genet. 2018 Aug 17;19:60. doi: 10.1186/s12863-018-0661-4 (PMC6098602; doi:10.1186/s12863-018-0661-4)
Supplement: Supplementary file 1 — Appendix 1. Derivation of third order disequilibrium in population admixture. (DOCX 19 kb) [file 12863_2018_661_MOESM1_ESM.docx]

**Appendix 1**

**Derivation of third order disequilibrium in population admixture**

From equation (4) in the body of the manuscript, third order linkage disequilibrium in the crossed population, Z, is:

$\delta_{TMK}^{Z}= {f_{TMK}^{Z}-f}_{T}^{Z}f_{M}^{Z}f_{K}^{Z}-f_{T}^{Z}\delta_{MK}^{Z}-f_{M}^{Z}\delta_{TK}^{Z}-f_{K}^{Z}\delta_{TM}^{Z}$.

Substituting $f_{TMK}^{Z}= \tau f_{TMK}^{X}+(1-\tau) f_{TMK}^{Y}$, the disequilibrium becomes

$\delta_{TMK}^{Z}= {\tau f}_{TMK}^{X}+(1-\tau) f_{TMK}^{Y}{-f}_{T}^{Z}f_{M}^{Z}f_{K}^{Z}-f_{T}^{Z}\delta_{MK}^{Z}-f_{M}^{Z}\delta_{TK}^{Z}-f_{K}^{Z}\delta_{TM}^{Z}$.

Substituting $f_{TMK}^{X}= f_{T}^{X}f_{M}^{X}f_{K}^{X}+f_{T}^{X}\delta_{MK}^{X}+f_{M}^{X}\delta_{TK}^{X}+f_{K}^{X}\delta_{TM}^{X}+\delta_{TMK}^{X},$and $f_{TMK}^{Y}= f_{T}^{Y}f_{M}^{Y}f_{K}^{Y}+f_{T}^{Y}\delta_{MK}^{Y}+f_{M}^{Y}\delta_{TK}^{Y}+f_{K}^{Y}\delta_{TM}^{Y}+\delta_{TMK}^{Y}$ into the above equation yields:

$$\delta_{TMK}^{Z}= \tau(f_{T}^{X}f_{M}^{X}f_{K}^{X}+f_{T}^{X}\delta_{MK}^{X}+f_{M}^{X}\delta_{TK}^{X}+f_{K}^{X}\delta_{TM}^{X}+\delta_{TMK}^{X})+(1-\tau) (f_{T}^{Y}f_{M}^{Y}f_{K}^{Y}+f_{T}^{Y}\delta_{MK}^{Y}+f_{M}^{Y}\delta_{TK}^{Y}+f_{K}^{Y}\delta_{TM}^{Y}+\delta_{TMK}^{Y} ){-f}_{T}^{Z}f_{M}^{Z}f_{K}^{Z}-f_{T}^{Z}\delta_{MK}^{Z}-f_{M}^{Z}\delta_{TK}^{Z}-f_{K}^{Z}\delta_{TM}^{Z}$$

Substituting allele frequencies $f_{T}^{Z}= \tau f_{T}^{X}+(1-\tau) f_{T}^{Y}$, $f_{M}^{Z}= \tau f_{M}^{X}+(1-\tau) f_{M}^{Y}$, and $f_{T}^{Z}= \tau f_{T}^{X}+(1-\tau) f_{T}^{Y}$ into the above equation, the disequilibrium becomes

$$\delta_{TMK}^{Z}= \tau(f_{T}^{X}f_{M}^{X}f_{K}^{X}+f_{T}^{X}\delta_{MK}^{X}+f_{M}^{X}\delta_{TK}^{X}+f_{K}^{X}\delta_{TM}^{X}+\delta_{TMK}^{X})+(1-\tau) (f_{T}^{Y}f_{M}^{Y}f_{K}^{Y}+f_{T}^{Y}\delta_{MK}^{Y}+f_{M}^{Y}\delta_{TK}^{Y}+f_{K}^{Y}\delta_{TM}^{Y}+\delta_{TMK}^{Y} )- (\tau f_{T}^{X}+(1-\tau) f_{T}^{Y})( \tau f_{M}^{X}+(1-\tau) f_{M}^{Y})( \tau f_{T}^{X}+(1-\tau) f_{T}^{Y})-f_{T}^{Z}\delta_{MK}^{Z}-f_{M}^{Z}\delta_{TK}^{Z}-f_{K}^{Z}\delta_{TM}^{Z}$$

Chakraborty and Smouse [13] expressed second order linkage disequilibrium in the cross as a function of the disequilibria and the differences in allele frequency at the two populations being crossed. Thus, $\delta_{MK}^{Z}=\tau\delta_{MK}^{X}+(1-\tau)\delta_{MK}^{Y}+\tau(1-\tau)(\gamma_{M}\gamma_{K})$, $\delta_{TK}^{Z}=\tau\delta_{TK}^{X}+(1-\tau)\delta_{TK}^{Y}+\tau(1-\tau)(\gamma_{T}\gamma_{K})$, and $\delta_{TM}^{Z}=\tau\delta_{TM}^{X}+(1-\tau)\delta_{TM}^{Y}+\tau(1-\tau)(\gamma_{T}\gamma_{M})$, where $\gamma_{T}$, $\gamma_{M}$, and $\gamma_{K}$ represent the differences in allele frequency in the two populations at crossing for loci *T/t*, *M/m*, and *K/k*, respectively. Substituting, these second order coefficients and allele frequencies $f_{T}^{Z}$, $f_{M}^{Z}$, and $f_{K}^{Z}$, the third order linkage disequilibrium of the admixture population is:

$$\delta_{TMK}^{Z}=\tau f_{T}^{X}f_{M}^{X}f_{K}^{X} +\tau f_{T}^{X}\delta_{MK}^{X}+{\tau f}_{M}^{X}\delta_{TK}^{X}+{\tau f}_{K}^{X}\delta_{TM}^{X}+{\tau\delta}_{TMK}^{X}$$

$+(1-\tau)f_{T}^{Y}f_{M}^{Y}f_{K}^{Y} +(1-\tau)f_{T}^{Y}\delta_{MK}^{Y}+{(1-\tau)f}_{M}^{Y}\delta_{TK}^{Y}+(1-{\tau)f}_{K}^{Y}\delta_{TM}^{Y}+{(1-\tau)\delta}_{TMK}^{Y}$

$-(\tau f_{T}^{X}+(1-\tau)f_{T}^{Y})(\tau f_{M}^{X}+(1-\tau)f_{M}^{Y})(\tau f_{K}^{X}+(1-\tau)f_{K}^{Y})$

$-(\tau f_{T}^{X}+(1-\tau)f_{T}^{Y}) (\tau\delta_{MK}^{X}+(1-\tau)\delta_{MK}^{Y}+\tau(1-\tau)(\gamma_{M}\gamma_{K}))$

$-(\tau f_{M}^{X}+(1-\tau)f_{M}^{Y}) (\tau\delta_{TK}^{X}+(1-\tau)\delta_{TK}^{Y}+\tau(1-\tau)(\gamma_{T}\gamma_{K}))$

$-(\tau f_{K}^{X}+(1-\tau)f_{K}^{Y}) (\tau\delta_{TM}^{X}+(1-\tau)\delta_{TM}^{Y}+\tau(1-\tau)(\gamma_{T}\gamma_{M}))$.

This equation illustrates that third order linkage disequilibrium in the crossed population just depends on allele frequencies, and second and third order linkage disequilibrium in the two populations at crossing.
