# Supplementary material for: Extent of third-order linkage disequilibrium in a composite line of Iberian pigs
Source: BMC Genet. 2018 Aug 17;19:60. doi: 10.1186/s12863-018-0661-4 (PMC6098602; doi:10.1186/s12863-018-0661-4)
Supplement: Supplementary file 2 — Appendix 2. Subroutine to estimate third order linkage disequilibrium. (DOCX 16 kb) [file 12863_2018_661_MOESM2_ESM.docx]

**Appendix 2**

**Subroutine to estimate third order linkage disequilibrium**

subroutine em3 (nn,f,nconv)

c nn is a matrix with 3 dimensions containing genotypic counts for the 3 loci

c f is a vector with estimate of 8 haplotype frequencies)

c nconv scalar for convergence value 1 if not

integer e1,e2,e3,nconv

parameter (nmar=10000)

c number maximum of markers

real*16 f(2,2,2),fa(2),fb(2),fc(2),fklm

real*16 ftotal(2,2,2)

integer u1(3), u2(3),u3(3)

integer nn(3,3,3),nnn

real*16 lik, dif

nconv=0

c estimating allele frequencies

do 32 l=1,3

do 32 m=1,3

rtot=nn(1,l,m)+nn(2,l,m)+nn(3,l,m)+rtot

fa(1)=nn(1,l,m)+0.5*nn(3,l,m)

32 continue

fa(1)=fa(1)/rtot

fa(2)=1.-fa(1)

c print*,rtot,fa(1)

rtot=0

do 33 k=1,3

do 33 m=1,3

rtot=nn(k,1,m)+nn(k,2,m)+nn(k,3,m)+rtot

fb(1)=nn(k,1,m)+0.5*nn(k,3,m)

33 continue

fb(1)=fb(1)/rtot

fb(2)=1.-fb(1)

rtot=0

do 34 k=1,3

do 34 l=1,3

rtot=nn(k,l,1)+nn(k,l,2)+nn(k,l,3)+rtot

fc(1)=nn(k,l,1)+0.5*nn(k,l,3)

34 continue

fc(1)=fc(1)/rtot

fc(2)=1.-fc(1)

c estimating haplotype frequencies at equilibrium

f=0

do 334 k=1,2

do 334 l=1,2

do 334 m=1,2

f(k,l,m)=fa(k)*fb(l)*fc(m)

c print*,' initial ', k,l,m,f(k,l,m),fa(k),fb(l),fc(m)

334 continue

ite=0

c computing complementary haplotypes

333 continue

do 25 k=1,2

do 25 l=1,2

do 25 m=1,2

if (m.eq.1) then

e3=2

else

e3=1

endif

if (l.eq.1) then

e2=2

else

e2=1

endif

if (k.eq.1) then

e1=2

else

e1=1

endif

c EM algorith iterations

fklm=0

fklm=2.*nn(k,l,m)+nn(3,l,m)+nn(k,3,m)+nn(k,l,3)

c print*,fklm

if ((f(k,l,m)*f(e1,e2,m)+f(e1,l,m)*f(k,e2,m)).gt.0) then

fklm=fklm+nn(3,3,m)

%*((f(k,l,m)*f(e1,e2,m))/(f(k,l,m)*f(e1,e2,m)+f(e1,l,m)*f(k,e2,m)))

endif

if ((f(k,l,m)*f(e1,l,e3)+f(e1,l,m)*f(k,l,e3)).gt.0) then

fklm=fklm+nn(3,l,3)*

%(f(k,l,m)*f(e1,l,e3)/(f(k,l,m)*f(e1,l,e3)+f(e1,l,m)*f(k,l,e3)))

endif

if ((f(k,l,m)*f(k,e2,e3)+f(k,e2,m)*f(k,l,e3)).gt.0) then

fklm=fklm+nn(k,3,3)*

%(f(k,l,m)*f(k,e2,e3)/(f(k,l,m)*f(k,e2,e3)+f(k,e2,m)*f(k,l,e3)))

endif

if((f(k,l,m)*f(e1,e2,e3)+f(e1,e2,m)*f(k,l,e3)

%+f(e1,l,m)*f(k,e2,e3)+f(e1,l,e3)*f(k,e2,m)).gt.0) then

fklm=fklm+nn(3,3,3)

%*(f(k,l,m)*f(e1,e2,e3)/(f(k,l,m)*f(e1,e2,e3)+f(e1,e2,m)*f(k,l,e3)

%+f(e1,l,m)*f(k,e2,e3)+f(e1,l,e3)*f(k,e2,m)))

endif

f(k,l,m)=fklm/(2.*rtot)

25 continue

ite=ite+1

c write(88, *)ite,f,dif

c write(88, *) ' '

c print*, ite,f(1,1,1),nn(1,1,1)

if (ite.eq.1) then

do 50 i1=1,2

do 50 i2=1,2

do 50 i3=1,2

ftotal(i1,i2,i3)=f(i1,i2,i3)

50 continue

ftotal=f

go to 333

endif

dif=0

if (ite.gt.1) then

do 55 i1=1,2

do 55 i2=1,2

do 55 i3=1,2

dif=dif+abs(f(i1,i2,i3)-ftotal(i1,i2,i3))

55 continue

endif

c print*,dif

if (dif.lt..000000001) then

do 54 i1=1,2

do 54 i2=1,2

do 54 i3=1,2

c print*, ite,f(i1,i2,i3),dif

c dif: difference between to iteracions: convergence criterium

54 continue

return

endif

if (ite.gt.100000) then

print*,' NO CONVERGENCE AT ITER ',ite,dif,ftotal

stop

nconv=1

return

endif

ftotal=f

go to 333

end
